# Efficacy of Hemiarthroplasty vs. Locking Plate Fixation for Proximal Humerus Fractures: A Meta-Analysis

**DOI:** 10.3389/fsurg.2021.651554

**Published:** 2021-09-21

**Authors:** Jiali Deng, Shuai Zhang, Yuanyuan Yu, Li Zhang, Li Zhang, Wen Jiang, Kai Yang, Xiaoyan Xi

**Affiliations:** ^1^Department of Orthopedics, Clinical Medical College and The First Affiliated Hospital of Chengdu Medical College, Sichuan, China; ^2^Department of Anesthesiology, Clinical Medical College and The First Affiliated Hospital of Chengdu Medical College, Sichuan, China; ^3^Department of General Medicine, The Third People's Hospital of Chengdu, Sichuan, China; ^4^Emergency and Business Management Office, Chengdu Center for Disease Control and Prevention, Sichuan, China

**Keywords:** hemiarthroplasty, proximal humerus fractures, meta-analysis, locking plate fixation, functional recovery

## Abstract

**Background:** Proximal humerus fractures are common in a clinic and account for ~6% of all adult fractures. Hemiarthroplasty (HA) or locking plate (LP) fixation is currently recommended for the treatment of complex proximal humerus fractures (PHFs); however, there is no uniform standard for optimal surgical treatment or functional recovery. We conducted a meta-analysis to compare the efficacy of LP and HA in the treatment of PHFs.

**Methods:** Relative studies associated with HA and LP were searched in December 2020 in the PubMed, Embase, Cochrane Library, and OVID databases. The quality of the studies, functional outcomes (including the Constant-Murley score (CMS), American Shoulder and Elbow Surgeons Score (ASES), Simple Shoulder Test (SST), Short Form Health Survey (SF-12v2), complications, and reoperation rate were extracted and analyzed with the Stata 14.0 software.

**Results:** A total of 958 patients from 12 studies were included in the meta-analysis, which showed that patients treated with LP had a significantly lower reoperation rate, a higher complication rate, and a higher CMS score than those treated with HA. There were no significant differences in ASES, SST, or SF-12v2 scores between treatment groups.

**Conclusions:** Compared with HA, LP exhibited better clinical efficacy in some aspects. However, large sample and randomized, controlled studies are needed for further validation.

## Introduction

Proximal humerus fractures are fractures that are usually attributed to osteoporosis (1) and are mostly caused by low-energy trauma (2, 3). It is a common clinical problem and accounts for ~6% of all adult fractures (4). The incidence of proximal humerus fractures (PHFs) in elderly patients is second only to the incidence of hip and distal radius fractures; thus, PHFs have become a serious public health problem (5, 6). The ideal treatment for PHFs remains controversial. Conservative measures and/or surgical management are still considered as the mainstays of treatment (7). Among them, open reduction and internal fixation (ORIF), hemiarthroplasty (HA), and reverse shoulder arthroplasty (RSA) are the primary methods of surgical treatment (8).

Although there are many surgical methods for PHF, there is still no unified standard for the selection of surgical methods. Therefore, the choice of surgical method depends mostly on the experience and personal preference of the surgeon (9). In recent decades, HA has become the treatment of choice for severe comminuted and displaced fractures of PHFs. However, postoperative joint function was not satisfactory. There were relatively high postoperative complications (10), and the rate of nonunion of large tubercles was as high as 17% (11, 12). Currently, the development of internal fixation technology, especially the appearance of locking plates (LPs), has provided a new idea for the treatment of PHFs (13, 14). However, despite the improvement of fixation techniques may still be inaccurate. The risk of losing fixation, nonunion, or ischemic necrosis is considered too high to attempt internal fixation (15, 16).

Although LP or HA is currently recommended for the treatment of complex PHFs, there remains some controversy. In addition, there is no uniform standard for optimal surgical treatment or functional recovery. To date, only a few related randomized clinical trials (RCTs) have been carried out, and some reported systematic reviews could not provide convincing evidence for clinical decision-making (17). In this study, we intended to explore the differences in complications and efficacy between LP and HA in the treatment of PHFs by meta-analysis to provide a reference for the selection of surgical methods for PHFs.

## Methods

### Search Strategy

Two of the authors independently searched the PubMed, Cochrane Library, Embase, and OVID electronic literature databases in December 2020. A combination of different terms and synonyms of keywords was used as follows: proximal humeral fracture, shoulder fracture, locking plate, plate fixation, plate internal fixation, PHILOS plate, locking compression plate, arthroplasty, joint replacement, and hemiarthroplasty. Additionally, through the search of references from previously published randomized trials, reviews, and meta-analyses for additional eligible studies, relevant articles, and reference lists were searched to avoid original omissions.

### Eligibility Criteria

#### Inclusion Criteria

(1) Randomized or nonrandomized controlled trials (evidence at or above level III); (2) patients with Neer II to IV fractures who were treated with LP and HA; (3) patients with a follow-up term of at least 3 months, functional outcomes, complications, and reoperation rate were used as results of the evaluation; and (4) relevant outcome indicators were included in the research results, and reliable data could be extracted from the full text.

#### Exclusion Criteria

(1) Other treatment methods, such as nonsurgical treatment, intramuscular nail fixation, and reverse shoulder arthroplasty; (2) exclusion of pathological fractures, multiple nonproximal humeral fractures, old fractures, etc.; (3) case report, systematic review, and animal experiments; (4) articles that fail to extract valid data and evidence lower than level III; and (5) without fulltext.

### Data Extraction

Two reviewers extracted the data according to the inclusion and exclusion criteria independently, and quality evaluation and data extraction were also carried out. If there was a disagreement, a further decision was adjudicated by the third author. The extracted data included the following: authors, publication date, research type/evidence level, sample size, fracture classification, follow-up term, etc. The effectiveness evaluation indices included functional outcomes [including the Constant-Murley score (CMS), American Shoulder and Elbow Surgeons Score (ASES), Simple Shoulder Test (SST), Short Form Health Survey (SF-12v2)], complications, and reoperation rate. For continuous outcomes, the mean and standard deviation (SD) and participant numbers were extracted. If these data were not available, the data were calculated using the methods described by Hozo (18).

### Statistical Analysis

Bicategorized index data were calculated by the odds ratio (OR) and 95% confidence intervals (CI), and the combined odds ratio (pooled OR) was calculated using the Mantel–Haenszel model. Continuity index data were calculated using the standardized mean difference (SMD) and 95% CI, and the combined SMD was determined using the random or fixed effects model. The heterogeneity between documents was determined by calculating *Q* and *I*^2^ statistics. If the heterogeneity (*I*^2^) > 20%, the results were combined with a random effects model; otherwise, a fixed effects model was used. Publishing bias was calculated with the Begg method. All the data were collected and analyzed using the Stata 14.0 software (Stata, College Station, TX, United States). *P* < 0.05 was considered to be statistically significant.

## Results

### Included Studies

**Figure 1:**
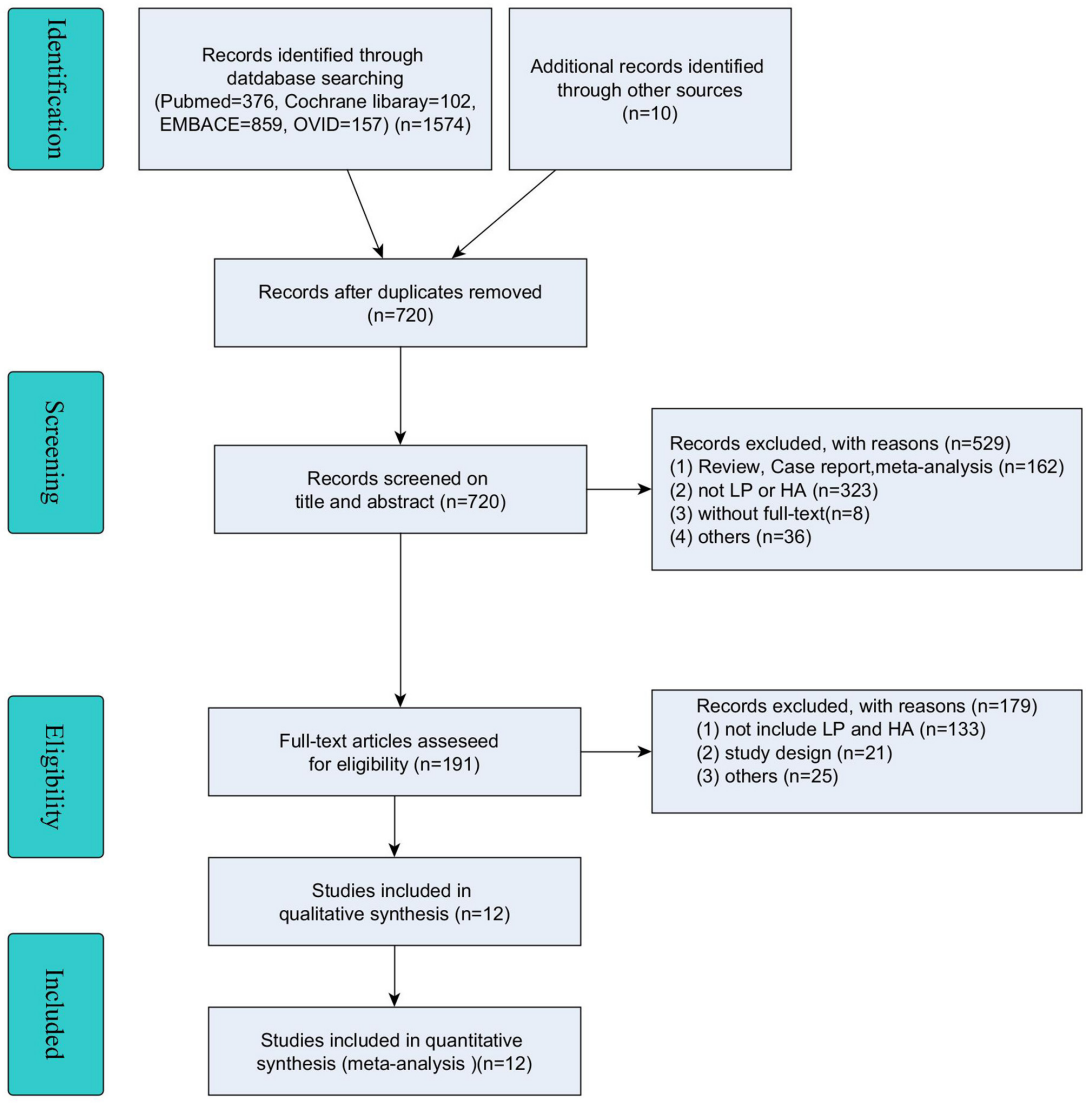
Flowchart of study selection.

A total of 1,574 potential references were identified through PubMed (*n* = 376), Cochrane Library (*n* = 102), Embase (*n* = 859), and OVID (*n*=237), and another 10 records were identified through other sources. After the removal of duplicates (*n* = 854), 720 articles were screened for relevance based on the title and abstract. Finally, 191 articles were eligible for inclusion, of which 179 were excluded for reasons of “without fulltext”, “not treated with LP and HA” and some other reasons. The remaining 12 studies were included in this meta-analysis (19–30) (details are shown in Figure 1).

### Characteristics and Qualifications of Included Studies

The characteristics of all the 12 included articles are summarized and shown in Table 1. Of all the included articles, one was a randomized controlled trial (RCT), two were prospective studies (PROs), and nine were retrospective case-control studies (CCSs), which were from four different countries (five from China, four from the United States, two from Switzerland, and one from Italy). A total of 613 patients in the LP group and 345 patients in the HA group were included in this meta-analysis. The article quality assessment and main observation indices are presented in Tables 2, 3, respectively.

**Table 1:** Characteristics of the included studies.

**References**	**Country**	**Study design/Evidence level**	**Age**	**Gender (M/F)**		**Fracture type**	**Follow-up time (months)**	**Outcome**
				**HA**	**LP**			
Solberg et al. (19)	America	CCS/ III	67.7	48	38	Neer III-IV	29	NR
Bastian and Hertel (22)	Switzerland	Pro/ III	60	33	44	Neer II-IV	56	HA
Wang et al. (27)	China	CCS/ III	49	10	12	Neer III-IV	20	NR
Zhang et al. (28)	China	CCS/ III	67.7	30	28	Neer III-IV	28	NR
Wild et al. (24)	America	CCS/ III	56.9	1/14	14/28	Neer III-IV	35.4	LP
Spross et al. (20)	Switzerland	CCS/ III	75.2	3/19	4/18	Neer VI	83	HA
Cai et al. (23)	China	RCT/ III	71.9	3/16	2/11	Neer IV	24	HA
Lu and TM (29)	China	CCS/ III	67	22	26	Neer IV	>6	NR
Chalmers et al. (21)	America	CCS/ III	H72/L71	9	9	Neer III-IV	12	HA
Thorsness et al. (30)	America	CCS/ III	H69.3/L64	17/66	105/225	Neer III-IV	/	NR
Chen et al. (25)	China	Pro / III	H64/L68	30	30	Neer IV	24	LP
Repetto et al. (26)	Italy	CCS/ III	67.7	24	19	Neer III-IV	39.7	NR

*RCT, randomized controlled trial; CCS, retrospective case-control study; Pro, prospective study; HA, hemiarthroplasty; LP, locking plate fixation; NR, neutral; M/F, male/female*.

**Table 2:** Methodological quality assessment of included studies.

**Indexes**	**Solberg et al. (19)**	**Bastian and Hertel (22)**	**Wang et al. (27)**	**Zhang et al. (28)**	**Wild et al. (24)**	**Spross et al. (20)**	**Cai et al. (23)**	**Lu and TM (29)**	**Thorsness et al. (30)**	**Chalmers et al. (21)**	**Chen et al. (25)**	**Repetto et al. (26)**
Representation of exposed queues	Y	Y	Y	Y	Y	Y	Y	Y	Y	Y	Y	Y
Selection of non-exposed queues	Y	Y	Y	Y	Y	Y	Y	Y	Y	Y	Y	Y
Exposure determination	Y	Y	Y	Y	Y	Y	Y	Y	Y	Y	Y	Y
No subjects at the beginning of the study	Y	Y	Y	Y	Y	Y	Y	Y	Y	Y	Y	Y
Diseases already occurred	N	N	N	N	N	N	N	N	N	N	N	N
Comparability of exposed and non-exposed queues	Y	N	N	Y	N	Y	N	N	N	N	N	Y
Methods of results determination	Y	Y	Y	N	N	Y	N	Y	N	Y	N	Y
Follow-up time long enough	Y	Y	Y	Y	Y	Y	Y	N	Y	Y	Y	Y
Follow-up integrity	Y	Y	Y	Y	Y	Y	Y	Y	Y	Y	Y	Y
Total (NOS)	8	7	7	7	6	8	6	5	6	7	6	8

*The evaluation criteria for follow-up time was over 12 months, and follow-up integrity was > 80*.

**Table 3:** Main observation indexes.

**References**	**Complications**		**Reoperation rate**		**Functional indexes**							
					**CMS**		**ASES**		**SST**		**SF-12v2**	
	**HA**	**LP**	**HA**	**LP**	**HA**	**LP**	**HA**	**LP**	**HA**	**LP**	**HA**	**LP**
Solberg et al. (19)	10	19	8	11	60.65 ± 0.9	68.69 ± 0.5	/	/	/	/	/	/
Bastian and Hertel (22)	10	24	4	7	70.01 ± 1.25	77.01 ± 5.25	/	/	/	/	/	/
Wang et al. (27)	0	2	/	/	65.15 ± 0.3	69.66 ± 0.7	/	/	/	/	/	/
Zhang et al. (28)	3	3	/	/	85.55 ± 0.6	83.96 ± 0.8	/	/	/	/	/	/
Wild et al. (24)	2	3	1	3	44.82 ± 2.6	70.12 ± 1.8	56.92 ± 0.3	71.62 ± 1.0	4.82 ± 0.7	7.63 ± 0.8	35.88 ± 0.3	40.81 ± 0.9
Spross et al. (20)	17	14	1	10	54.41 ± 0.0	65.21 ± 7.0	/	/	/	/	/	/
Cai et al. (23)	1	1	3	3	72.9	60.7	/	/	/	/	/	/
Lu and TM (29)	3	3	/	/	70.41 ± 2.4	73.61 ± 1.0	/	/	/	/	/	/
Chalmers et al. (21)	7	23	/	/	/	/	/	/	/	/	/	/
Thorsness et al. (30)	1	1	/	/	/	/	66 ± 31	75 ± 15	7 ± 4	8 ± 4	34 ± 7	34 ± 6
Chen et al. (25)	/	/	/	/	75.46 ± 0.2	80.11 ± 0.2	/	/	/	/	/	/
Repetto et al. (26)	/	/	/	/	48.42 ± 7.3	61.81 ± 4.7	/	/	5.63 ± 0.9	11.73 ± 0.1	/	/

### Comparison of Complications

**Figure 2:**
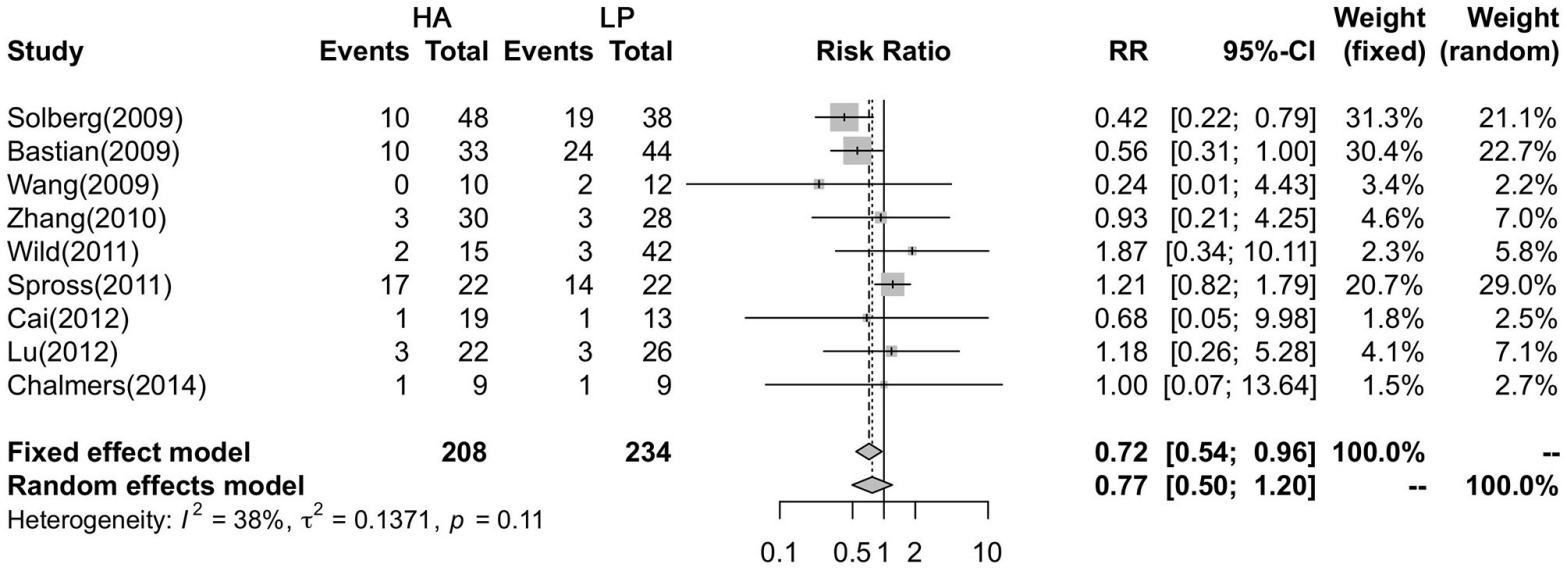
Forest plot showing the meta-analysis of the complications.

Nine studies reported that the complication rate, with a total of 147 patients (54 in the HA group and 93 in the LP group), was analyzed with a fixed effects model and showed no heterogeneity in each study (*P* = 0.115, *I*^2^= 38.1%). The results showed that the LP group had a higher postoperative complication rate than the HA group (29.91 vs. 22.6%, *P* = 0.024) (Figure 2).

### Comparison of Reoperation Rate

**Figure 3:**
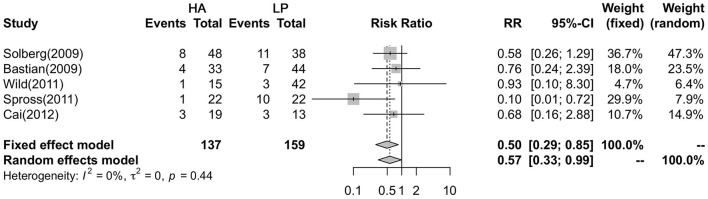
Forest plot showing the meta-analysis of the reoperation rate.

A fixed effects model was utilized to analyze the differences in reoperation rates in five studies. The results showed that there was no heterogeneity among the five studies (*P* = 0.443, *I*^2^ = 0%). In addition, the results showed a higher reoperation rate in the HA group than in the LP group (*RR* = 0.495, 95% CI:0.289–0.848; *P* = 0.01) (Figure 3).

### Comparison of Functional Outcomes

**Figure 4:**
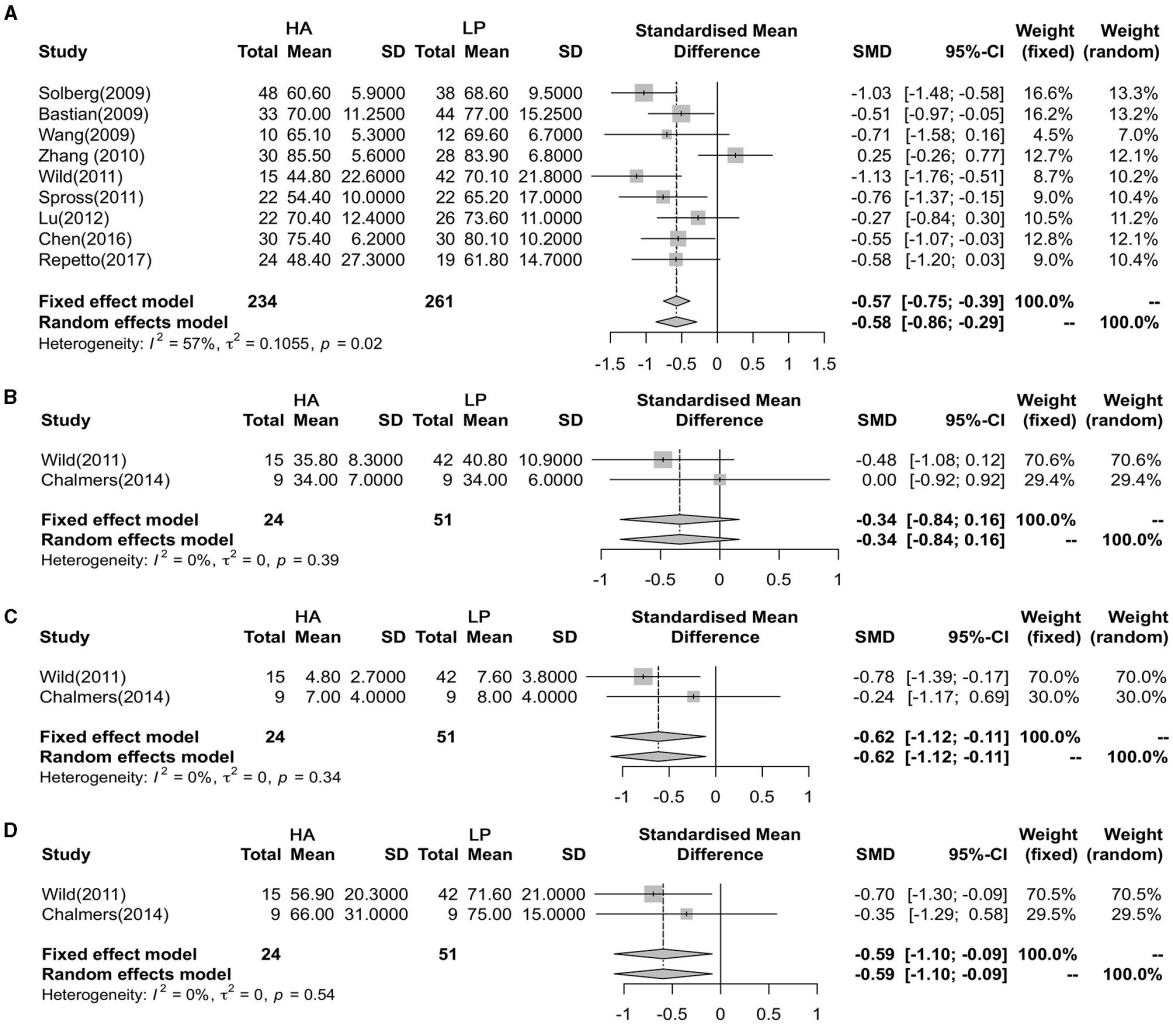
Forest plot showing the meta-analysis of the functional outcome. **(A)** Constant-Murley score (CMS); **(B)** Short Form Health Survey (SF-12V2); **(C)** Simple Shoulder Test (SST); **(D)** American Shoulder and Elbow Surgeons Score (ASES).

The CMS, ASES, SF-12V2, and SST were used to estimate the functional outcome of the two groups. The meta-analysis showed no heterogeneity among the nine studies (*P* = 0.015, *I*^2^ = 57.7%), and the CMS in the LP group was better than that in the HA group (SMD = −0.585, 95% CI: −0.874 to −0.296; *P* < 0.001) (Figure 4A). In addition, the meta-analysis showed that there were significant differences between ASES (95% CI: −1.114 to −0.099; *P* =0.019) and SST (95% CI: −1.135 to −0.118; *P* = 0.016), without heterogeneity (*P* = 0.553, *I*^2^ = 0%; *P* = 0.342, *I*^2^ = 0%; respectively), and that there were no differences in SF-12V2 (95% CI: −0.844 to −0.159; *P* = 0.18) (Figures 4B–D).

### Publication Bias Recognition

**Figure 5:**
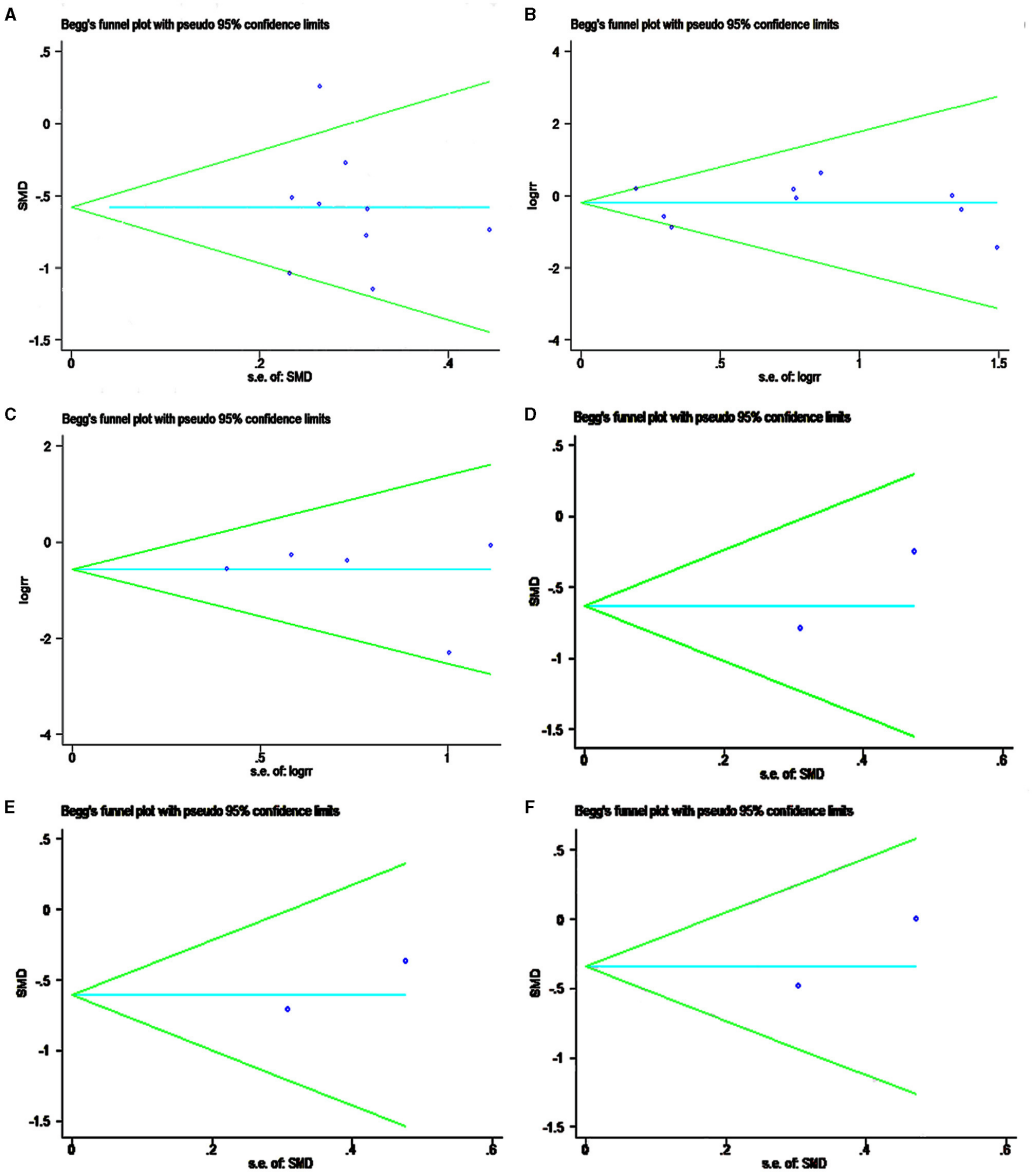
Funnel plot showing the meta-analysis of publication bias recognition. **(A)** CMS; **(B)** complications; **(C)** reoperation rate; **(D)** SST; **(E)** ASES; **(F)** SF-12V2.

Publication bias analysis showed that the complications, reoperation rate, and CMS of Begg's test funnel plot statistics were 0.466, 1.000, and 0.602, respectively (Figures 5A–C). Almost all the scattered studies were located in a funnel map, suggesting that the publication bias of the included studies was small (Figure 5).

## Discussion

As an important functional unit of the body, shoulder joint function will be seriously affected after trauma, and reasonable treatment seems important. To date, the treatment of PHFs is still a controversial topic and a major challenge for surgeons. In particular, complex fracture patterns usually lead to pain and functional patterns, which lead many clinicians to regard three- or four-part fractures as indications for surgical treatment (10). However, whether the surgical method can achieve a satisfactory shoulder joint function is still controversial (31–33). The meta-analysis from the selected literature showed that LP has more advantages in the evaluation of postoperative function, and that the literature was heterogeneous.

The CMS, ASES, SST and SF-12v2 were used extensively for clinical evaluation standards for shoulder joints (34); the higher the score, the better the shoulder function. The use of CMS helps to perceive the diversity of consequences, especially the power of evaluation and scope of activity, which aroused the concern of examiners on the lack of evaluation standards and potential non-observer bias. Previous reports have shown that the CMS among female patients over 60 years old was 69–70, and that it was 75–83 in male patients (35). In this study, we found a significant difference in the total CMS, and ASES and SST scores between the LP and HA groups. After operation, all patients in both groups obtained satisfactory shoulder function.

The incidence and types of complications vary from one document to another, such as fracture nonunion, dislocation, infection, avascular necrosis (AVN), internal fixation, or prosthesis loosening or rupture (36, 37). In this study, the total postoperative complication rate of the included cases was analyzed, and the results showed a higher complication rate in the LP group (29.91% vs. 22.6%). In line with previous findings (38), this study showed an incidence of complications of 26.47%. AVN is an important complication of LP and occurs in 4 to 55% of humeral heads (19, 20). Due to the removal of humeral head in patients treated with HA, AVN may not occur after surgery. From a clinical perspective, the relative risk (RR) of AVN in the LP group was higher than that in the HA group. Additionally, the meta-analysis results also support this conclusion regarding the complications of loosening and displacement (data not shown).

Although complications were the cause of reoperation, not all of them were reoperated on. In the involved literature, Spross (20) considered the height of the prosthesis as a complication, but it did not undergo reoperation. This may explain why there was no difference in complications between the LP and HA groups, but a difference in the reoperation rate. However, because of different expectations of patients and/or physicians and possibly the exit of observation bias, reoperation rate has not been mentioned in some literature (21, 30). Inconsistent with some studies, the meta-analysis showed a higher reoperation rate in the HA group than in the LP group (39.71% vs. 25.94%), which suggested an advantage for the LP group.

There are several weaknesses in this study, which may affect the applicability of the conclusions. First, the methodological quality of published literature was insufficient, and the sample size of some literature was small, indicating inadequate statistical power. In addition, the simple method described by Hozo (18) was conducted to calculate the SD, which might cause a deviation in the results, but this deviation may decrease with increasing the sample size. In addition, the literature included in this article lacks long-term prospective randomized controlled trials. This is mainly due to the characteristics of clinical work, and it seems difficult to achieve a genuine randomized controlled trial. Multicenter, large-sample RCTs should be included to ensure that the results are more convincing. Additionally, a stratified analysis based on age, sex, level of motion, degree of fracture displacement, and degree of articular surface accumulation may compensate for the deficiency of this article. Moreover, the studies included in the meta-analysis were of a relatively poor level of evidence (level III), and more studies are needed.

## Conclusions

Through meta-analysis, this study determined the clinical effects of LP vs. HA on PHFs. The results showed that patients treated with LP exhibited better clinical efficacy in some aspects when compared with HA, which has the potential to help guide decision-making and weigh risks and benefits. However, limited by small sample size and RCT study, there is no consensus on which treatment is more suitable and advantageous.

## Data Availability Statement

The raw data supporting the conclusions of this article will be made available by the authors, without undue reservation.

## Author Contributions

JD and SZ conceived of the design of the study. YY, LZ (4th Author), and LZ (5th Author) performed the study, collected the data, and contributed to the design of the study. KY prepared the manuscript. XX edited the manuscript. All authors have read and approved the final manuscript.

## Conflict of Interest

The authors declare that the research was conducted in the absence of any commercial or financial relationships that could be construed as a potential conflict of interest.

## Publisher's Note

All claims expressed in this article are solely those of the authors and do not necessarily represent those of their affiliated organizations, or those of the publisher, the editors and the reviewers. Any product that may be evaluated in this article, or claim that may be made by its manufacturer, is not guaranteed or endorsed by the publisher.
